# A case report of myocardial infarction in a young transgender man with testosterone therapy: raising awareness on healthcare issues in the transgender community and a call for further research

**DOI:** 10.1093/ehjcr/ytad562

**Published:** 2023-12-06

**Authors:** Paul J Connelly, Joanna Osmanska, Matthew M Y Lee, Christian Delles, Margaret B McEntegart, John Byrne

**Affiliations:** School of Cardiovascular and Metabolic Health, University of Glasgow, Glasgow, UK; Department of Endocrinology and Diabetes, Queen Elizabeth University Hospital, Glasgow, UK; School of Cardiovascular and Metabolic Health, University of Glasgow, Glasgow, UK; Department of Endocrinology and Diabetes, Queen Elizabeth University Hospital, Glasgow, UK; School of Cardiovascular and Metabolic Health, University of Glasgow, Glasgow, UK; Department of Endocrinology and Diabetes, Queen Elizabeth University Hospital, Glasgow, UK; School of Cardiovascular and Metabolic Health, University of Glasgow, Glasgow, UK; Department of Endocrinology and Diabetes, Queen Elizabeth University Hospital, Glasgow, UK; Department of Endocrinology and Diabetes, Queen Elizabeth University Hospital, Glasgow, UK; West of Scotland Heart and Lung Centre, Golden Jubilee National Hospital, Glasgow, UK; Department of Cardiology, Columbia University Medical Center, New York, USA; Department of Endocrinology and Diabetes, Queen Elizabeth University Hospital, Glasgow, UK; West of Scotland Heart and Lung Centre, Golden Jubilee National Hospital, Glasgow, UK

**Keywords:** Case report, Acute coronary syndrome, Transgender, Testosterone

## Abstract

**Background:**

People who are transgender may utilize masculinizing or feminizing gender-affirming hormonal therapy. Testosterone and oestrogen receptors are expressed throughout the cardiovascular system, yet the effects of these therapies on cardiovascular risk and outcomes are largely unknown. We report the case of a young transgender man with no discernible cardiovascular risk factors presenting with an acute coronary syndrome.

**Case summary:**

A 31-year-old transgender man utilizing intramuscular testosterone masculinizing gender-affirming hormonal therapy presented with central chest pain radiating to the left arm. He had no past medical history of hypertension, dyslipidaemia, diabetes, or smoking. Electrocardiography demonstrated infero-septal ST depression, and high-sensitivity troponin-I was elevated and increased to 19 686 ng/L. He was diagnosed with a non–ST-segment elevation myocardial infarction. Inpatient coronary angiography confirmed a critical focal lesion in the mid right coronary artery, which was managed with two drug-eluting stents. Medical management (i.e. aspirin, ticagrelor, atorvastatin, ramipril, and bisoprolol) and surveillance of residual plaque disease evident in the long tubular left main stem, proximal left anterior descending, and proximal circumflex vessels was undertaken. The masculinizing gender-affirming hormonal therapy was continued.

**Discussion:**

Despite a greater awareness of the potential risk of increased cardiovascular disease in transgender people, the fundamental lack of data regarding cardiovascular outcomes in transgender people may be contributing to healthcare inequalities in this population. We must implement better training, awareness, and research into transgender cardiovascular health to facilitate equitable and evidence-based outcomes.

**Learning points**

- Transgender people experience gender dysphoria due to incongruence between their gender identity and the sex they were assigned at birth.
- Many transgender individuals utilize masculinizing (testosterone; transgender man) or feminizing (oestrogen; transgender women) gender-affirming hormone therapy.
- Sex hormones may modulate the development of cardiovascular disease; however, there is no consensus as to whether the risk of myocardial infarction is modified in transgender men utilizing testosterone.
- We must engage with individuals who are transgender in order to implement better clinical care and evidence-based guidance.

## Introduction

People who are transgender account for ∼0.5% of the general population, and the prevalence of this community is increasing.^1^ Transgender is a term for people whose gender identity and expression does not align with the sex they were assigned at birth. Therefore, transgender men were assigned as female at birth but identify and live their lives as men. Conversely, transgender women are assigned male sex at birth and identify as women. Individuals may also identify as non-binary, genderqueer, gender neutral, agender, gender fluid, and as a ‘third’ gender.^2^ Transgender individuals may engage with transmasculine (i.e. testosterone) or transfeminine (i.e. oestrogen ± androgen suppression therapies) gender-affirming hormone therapy to achieve secondary sex characteristic gender congruence.^3^

## Summary figure

**Table:** 

Timeline	
Day 0	7 p.m.: Onset of chest pain
Day 1	3 a.m.: Chest pain acutely worse
	4:16 a.m.: Called ambulance
	4:41 a.m.: Pre-hospital electrocardiogram (ECG) showed mild inferior ST elevation
	5:28 a.m.: Admitted to accident and emergency—medical therapy commenced
	6:48 a.m.: Electrocardiogram showed mild ST elevation in aVL and infero-posterior ST-segment depression—discussed with primary percutaneous coronary intervention (PCI) centre in a different hospital—delayed presentation and pain-free therefore not for urgent transfer
	8:16 a.m.: Electrocardiogram showed ST elevation in aVL resolved
	9:22 a.m.: Transferred to coronary care unit
Day 2	Formal transthoracic echocardiogram
Day 4	Transferred to tertiary cardiac centre
Day 5	Coronary angiogram and PCI
Day 6	Discharged from hospital

Recent evidence has suggested that the utilization of gender-affirming therapies may be associated with increased cardiovascular risk.^4^ Moreover, novel risk factors, such as minority stress (i.e. unique psychosocial stressors attributed to a minority identity), may also enhance this risk and cardiovascular disease burden within this population.^5^ Here, we present a case of a transgender man with a history of long-term testosterone usage, who has experienced an acute coronary syndrome at such a young age with no discernible traditional cardiovascular risk factors.

## Case presentation

A 31-year-old transgender man presented to the emergency department after an episode of central chest pain described as a constricting band radiating to his left arm, shoulder, and neck, associated with dyspnoea and paraesthesia of the hands. This came on at rest at 7 p.m. and self-terminated after 10 min but recurred intermittently. This acutely worsened at 3 a.m. the following morning. Emergency services were called at ∼4 a.m. and an ambulance brought him in to the hospital.

This transgender man (natal sex female) transitioned 4 years ago and had been on intramuscular Sustanon (testosterone esters) 125 mg every 3 weeks. Previous testosterone measurements were maintained within the male reference range. He had no other significant past medical history of note, including hypertension, dyslipidaemia, or diabetes. The patient never smoked, denied alcohol excess, and illicit substance misuse. There was no family history of heart disease.

His vital signs were unremarkable. Cardiovascular examination demonstrated normal heart sounds and non-elevated jugular venous pressure. Respiratory and abdominal examinations were normal. The patient was euvolaemic and his body mass index was 21.3 kg/m^2^.

**Figure 1:**
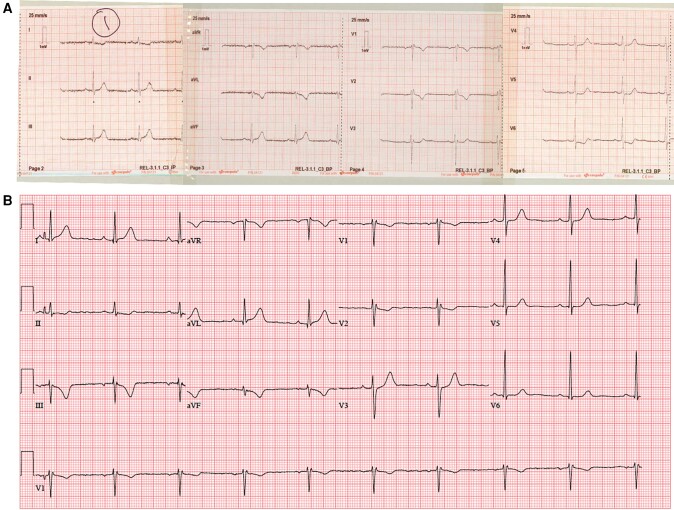

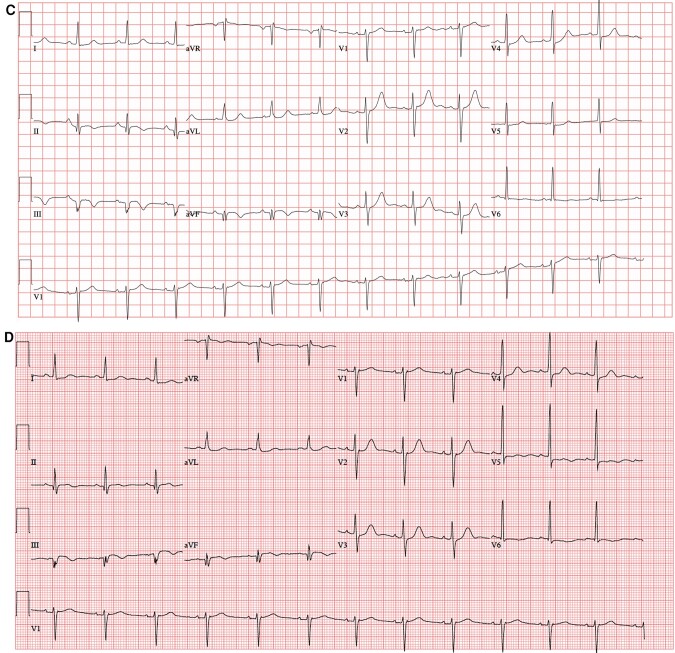
(*A*) Pre-hospital 12-lead electrocardiogram at 4:41 a.m. showing mild inferior ST elevation with lateral ST depression. (*B*) Emergency department 12-lead electrocardiography at 6:48 a.m. showing mild ST elevation in aVL and infero-posterior ST depression and T-wave inversion. (*C*) Repeat 12-lead electrocardiogram at 8:16 a.m. showing resolved ST elevation in aVL. (*D*) 12-lead electrocardiography performed 24 h post-admission showing new inferior Q waves.

The first pre-hospital 12-lead electrocardiogram (ECG) at 4:41 a.m. showed sinus rhythm with mild ST elevation inferiorly (leads II, III, and aVF) with ST depression laterally (leads I, aVL, and V5–V6) (*Figure 1A*). A repeat 12-lead ECG at 6:48 a.m. in the emergency department demonstrated mild ST elevation in lead aVL, infero-posterior ST depression, and T-wave inversion (*Figure 1B*). At the time, the tertiary cardiac referral centre for primary percutaneous coronary intervention (PCI) based in a different hospital was contacted and had advised that an urgent transfer was not indicated because of the delayed presentation and pain-free status. PR, QRS, and QTc intervals and axis were within normal limits. Further ECGs in the subsequent hours (*Figure 1C*) and following day showed development of inferior Q-waves (*Figure 1D*). Chest X-ray did not reveal any abnormality, and results of laboratory tests are shown in *Table 1*.

**Table 1:** Laboratory tests

Test	Value	Range	Units
High-sensitivity cardiac troponin-I	Day 1 (5:36 a.m.): 57 Day 1 (2:32 p.m.): 3817 Day 2 (6:00 a.m.): 19 686 Day 4 (5:44 a.m.): 11 302	0–34	ng/L
White blood cell count	14.6	4.0–10.0	×10^9^/L
Haemoglobin	152	130–180	g/L
Haematocrit	0.472	0.400–0.540	L/L
Mean corpuscular volume	85.4	83.0–101.0	fL
Platelet count	361	150–410	×10^9^/L
Sodium	138	133–146	mmol/L
Potassium	4.7	3.5–5.3	mmol/L
Urea	4.3	95–108	mmol/L
Creatinine	64	40–130	umol/L
Estimated glomerular filtration rate	>60	>60	mL/min/1.73m^2^
Bilirubin	11	<20	umol/L
Alanine transaminase	15	<50	U/L
Aspartate transaminase	26	<40	U/L
Alkaline phosphatase	109	30–130	U/L
Serum albumin	42	35–50	g/L
Total cholesterol	2.6		mmol/L
Triglycerides	1.2		mmol/L
Random blood glucose	5.5	3.5–6.0	mmol/L
Glycated haemoglobin	35	20–41	mmol/mol
C-reactive protein	9	0–10	mg/L
Testosterone	10.3	10.0–36.0	nmol/L
Sex hormone–binding globulin	47	13–70	nmol/L
Free testosterone	161	>200	pmol/L
SARS-CoV-2 PCR	Negative		

PCR, polymerase chain reaction; SARS-CoV-2, severe acute respiratory syndrome coronavirus 2.

Transthoracic echocardiography showed a non-dilated left ventricle with mild left ventricular systolic dysfunction (LVSD) with hypokinesis of the basal to mid infero-septal and infero-lateral segments (ejection fraction 48%) (see Supplementary material online, *Videos S1* and *S2*). The right ventricle was non-dilated with evidence of systolic dysfunction. The atria were not dilated, and there were no significant valvular abnormalities, shunts, or intracavity thrombus.

The working diagnosis was a delayed presentation inferior ST-segment elevation myocardial infarction (MI) vs. an inferior non–ST-segment elevation MI (NSTEMI). Initial treatments included dual-antiplatelet therapy loading with aspirin and ticagrelor and subcutaneous fondaparinux, in accordance with local acute coronary syndrome guidance.

**Figure 2:**
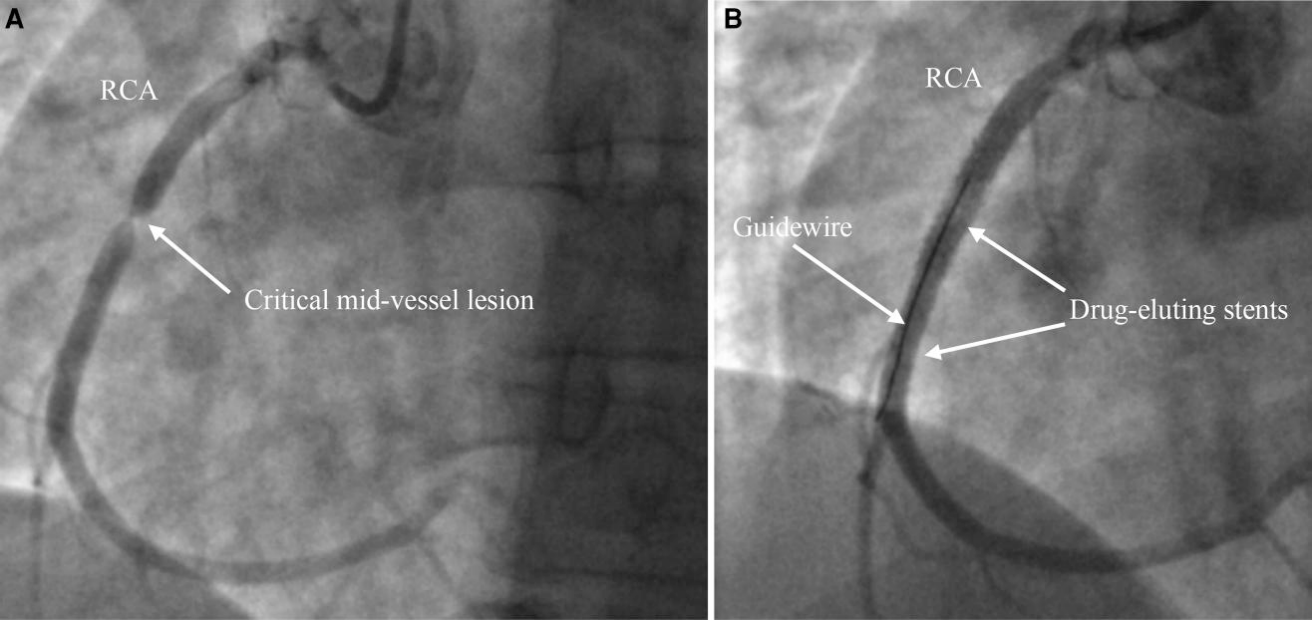
Coronary angiogram showing right coronary artery with critical stenosis mid-vessel pre-(*A*) and post-percutaneous coronary intervention (*B*) with two drug-eluting stents.

Subsequent urgent inpatient coronary angiography confirmed a critical focal lesion in the mid right coronary artery (RCA) (*Figure 2A*, Supplementary material online, *Video S3*) and long tubular left main stem (LMS) plaque disease, with disease in the proximal left anterior descending (LAD) and proximal circumflex (Cx) (see Supplementary material online, *Figure S1* and *Video S4*). A pressure wire study showed fractional flow reserve values of 0.80 in the LMS-LAD and 0.82 in the LMS-Cx. Percutaneous coronary intervention was performed to the mid RCA with two drug-eluting stents with an excellent result (*Figure 2B*). Medical management of residual LMS/LAD/Cx disease was recommended with a plan to consider surveillance for LMS disease via computed tomography coronary angiography.

He was discharged on Day 6, with medications including aspirin 75 mg once daily (lifelong), ticagrelor 90 mg twice daily (for 12 months), atorvastatin 80 mg once daily, ramipril 1.25 mg once daily, bisoprolol 2.5 mg twice daily, and glyceryl trinitrate spray sublingual as required. Follow-up was arranged in the post-MI LVSD pharmacist-led clinic and with cardiac rehabilitation. Testosterone therapy was continued for ongoing gender affirmation, as there is no guidance and insufficient evidence to support the discontinuation of gender-affirming hormone therapy or to switch to a transdermal formulation of this therapy. This patient moved to another country prior to follow-up, and therefore, these results cannot be provided.

## Discussion

Transgender men may seek out medical gender affirmation through masculinizing endocrine therapies, typically in the form of intramuscular or transdermal testosterone preparations.^6^ This therapy aims to promote and maintain the development of male secondary sex characteristics, to improve wellbeing and reduce gender dysphoria. Testosterone modulates the development of increased masculine hair growth, lean muscle mass and strength, clitoral enlargement, and the cessation of menses.^7^

However, androgen receptors are widely distributed and, in particular, are expressed throughout the vasculature in endothelial and vascular smooth muscle cells, which may result in unintended cardiovascular consequences.^8^

In cisgender men, whose natal sex is the same as their gender identity, retrospective analyses have demonstrated conflicting results of the use of exogenous testosterone on the risk of MI. In a cohort study of over 50 000 individuals who had received a testosterone prescription, the risk of MI following initiation of testosterone was significantly increased in older men and in younger men with pre-existing heart disease.^9^ However, in a cohort study of over 80 000 cisgender males, with a median age of 66 years, testosterone normalization by replacement therapy was associated with a significant reduction in all-cause mortality and MI.^10^ In a recent meta-analysis, including a total of 39 randomized control trials and 10 observational studies, exogenous testosterone treatment did not lead to a significant increase in the risk of MI; a major limitation of this assessment was the very low quality of evidence presented.^11^ Consequently, limited data are available for the risk of testosterone replacement and cardiovascular risk in younger individuals. Yet, at least one study has demonstrated, reassuringly, that the treatment of low testosterone in younger, healthy cisgender men was generally safe and not associated with an increased risk of MI.^12^

Due to the absence of good-quality data, it remains uncertain whether the risk of MI is modified in transgender men.^4,13^ Indeed, even confirming the effect of testosterone on blood pressure, a significant cardiovascular risk factor, in transgender men is not possible due to insufficient data and poor-quality evidence.^14^ Population-based surveys are limited by the absence of standardization, and no prospective or randomized studies relating to cardiovascular health in this population have been performed.^15^ A large retrospective cohort study of transgender people receiving testosterone could not draw meaningful conclusions on the risk of MI due to the young age of participants and small number of events.^16^ Larger cohorts with extended follow-up are required to study this issue which is vitally important to the health and wellbeing of transgender individuals.

Case reports of transgender men experiencing significant cardiovascular events are similarly limited. However, this case demonstrates striking comparisons to that published by Dinesh *et al.*^17^ who reported a 35-year-old transgender man with an acute inferior wall MI with a distal RCA occlusion. With the exception of a significant smoking history, this patient did not demonstrate other cardiovascular risk factors. In this case, there was no evidence of underlying coronary atherosclerosis, resulting in the conclusion that this lesion may have been cardioembolic in origin as a consequence of the prothrombotic effects of testosterone.

Moreover, Inoue *et al.*^18^ described a 32-year-old transgender man utilizing intramuscular testosterone who experienced a sudden unexpected death. Following autopsy, this was attributed to ischaemic heart disease with evidence of severe stenosis (>90%) within the left descending coronary branch. Consequently, these reports suggest transgender men using testosterone may be at risk of significant, and potentially life-threatening, cardiovascular events at a relatively young age in the absence of significant traditional cardiovascular risk factors. Consequently, it is imperative that we engage with the transgender community to undertake this vital research to provide them with safe and effective healthcare.

## Patient perspective

Our patient echoed literature reporting concerns regarding limited access to healthcare services and social stigmatization of transgender individuals, resulting in fear and reluctance to seek help.^19^ Studies show that waiting times for assessments and treatments for transgender individuals have not changed in the past 15 years, and 29% of the transgender population felt that being transgender adversely affected how healthcare professionals treated them.^19^ Transgender people report a perceived lack of knowledge and professional ignorance among clinicians, who may attribute physical symptoms to hormonal treatments even in the absence of robust evidence for this, affecting their access to routine treatments.^20^ Importantly, our patient had experienced chest pains for several years before starting testosterone that were not fully investigated; unfortunately, this lack of access to the usual clinical investigations might be a more common occurrence in gender-diverse individuals. Furthermore, the impact of mental health cannot be overlooked, as for some, the potential risk of hormone treatment is less important than the positive impact on their mental health.

Our patient recognized the importance of raising awareness of health risks in transgender people, including amongst healthcare professionals. He shared concerns that unsubstantiated misconceptions about the safety of gender-affirming hormone therapy might make it more challenging for transgender individuals to receive such treatments. This might lead to further inequalities in healthcare provision to transgender people.

In conclusion, healthcare professionals must receive adequate training on these crucial issues relating to the health of transgender people to help support decision-making, including the risks and benefits of treatments. However, this can only be facilitated through the inclusion of transgender individuals in cardiovascular research.

## Supplementary Material

ytad562_Supplementary_Data

## Data Availability

Anonymized data underlying this article will be shared upon reasonable request to the corresponding author.

## Lead author biography

Dr Paul J. Connelly is a clinical lecturer in endocrinology and diabetes at the School of Cardiovascular and Metabolic Health, University of Glasgow. He studied medicine and pharmacology at the University of Dundee before relocating to Glasgow to complete his clinical and academic training under the supervision of Professor Christian Delles. Dr Connelly’s research interests include cardiovascular endocrinology and the role of sex hormones in cardiovascular disease. He has a particular research focus in the cardiovascular health of people who are transgender.

## Supplementary material

**Consent:** The authors confirm that written consent for submission and publication of this case report including the images and associated text have been obtained from the patient in line with the Committee on Publication Ethics (COPE) guidance.

**Funding:** None declared.
