# Supplementary figures and images for: A case report of myocardial infarction in a young transgender man with testosterone therapy: raising awareness on healthcare issues in the transgender community and a call for further research
Source: Eur Heart J Case Rep. 2023 Dec 6;7(12):ytad562. doi: 10.1093/ehjcr/ytad562 (PMC10716680; doi:10.1093/ehjcr/ytad562)

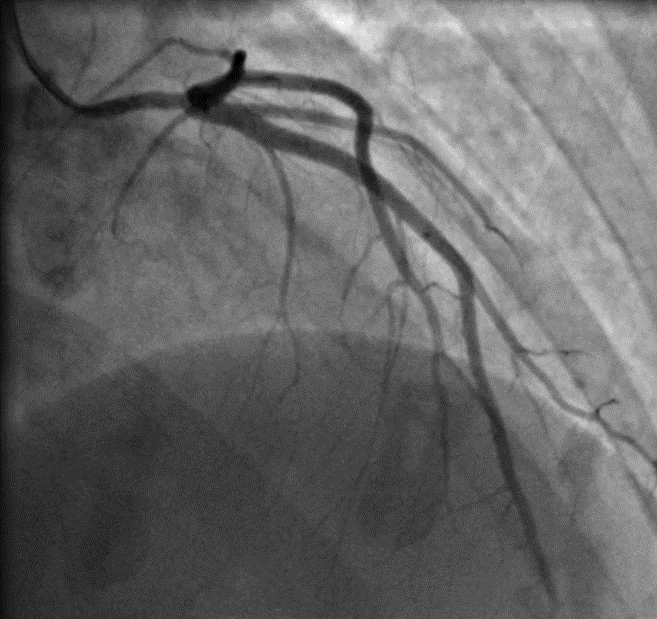

Supplement: ytad562_Supplementary_Data [file ytad562_supplementary_data.zip › Figure S1.png]
